# The Brazilian version of the Hip Sports Activity Scale: translation and cross-cultural adaptation

**DOI:** 10.1590/1516-3180.2021.0157.R1.23072021

**Published:** 2022-02-21

**Authors:** Letícia Nunes Carreras Del Castillo Mathias, Themis Moura Cardinot, Danúbia da Cunha de Sá-Caputo, Márcia Cristina Moura-Fernandes, Mário Bernardo-Filho, Gustavo Leporace de Oliveira Lomelino Soares, Luiz Alberto Batista, Liszt Palmeira de Oliveira

**Affiliations:** I MSc. Physiotherapist and Doctoral Student, Programa de Pós-Graduação em Ciências Médicas (PGCM), Universidade do Estado do Rio de Janeiro (UERJ), Rio de Janeiro (RJ), Brazil.; II PhD. Physical Educator and Professor, Departamento de Ciências Farmacêuticas (DCFar), Instituto de Ciências Biológicas e da Saúde (ICBS), Universidade Federal Rural do Rio de Janeiro (UFRRJ), Seropédica (RJ), Brazil.; III PhD. Physiotherapist and Researcher, Laboratório de Vibrações Mecânicas e Práticas Integrativas (LAVIMPI), Departamento de Biofísica e Biometria, Instituto de Biologia Roberto Alcântara Gomes, Policlínica Piquet Carneiro (PPC), Universidade do Estado do Rio de Janeiro (UERJ), Rio de Janeiro (RJ), Brazil.; IV MSc. Physiotherapist and Doctoral Student, Laboratório de Vibrações Mecânicas e Práticas Integrativas (LAVIMPI), Departamento de Biofísica e Biometria, Instituto de Biologia Roberto Alcântara Gomes, Policlínica Piquet Carneiro (PPC), Universidade do Estado do Rio de Janeiro (UERJ), Rio de Janeiro (RJ), Brazil.; V PhD. Physiotherapist and Professor, Laboratório de Vibrações Mecânicas e Práticas Integrativas (LAVIMPI), Departamento de Biofísica e Biometria, Instituto de Biologia Roberto Alcântara Gomes, Policlínica Piquet Carneiro (PPC), Universidade do Estado do Rio de Janeiro (UERJ), Rio de Janeiro (RJ), Brazil.; VI PhD. Physical Educator and Physiotherapist, Departamento de Diagnóstico por Imagem, Escola Paulista de Medicina, Universidade Federal de São Paulo (UNIFESP), São Paulo (SP), Brazil.; VII PhD. Physical Educator and Professor, Programa de Pós-Graduação em Ciências Médicas (PGCM), Universidade do Estado do Rio de Janeiro (UERJ), Rio de Janeiro (RJ), Brazil.; VIII MD, PhD. Orthopedist and Professor, Programa de Pós-Graduação em Ciências Médicas (PGCM), Universidade do Estado do Rio de Janeiro (UERJ), Rio de Janeiro (RJ), Brazil.

**Keywords:** Surveys and questionnaires, Femoroacetabular impingement, Sports, Translations, Hip injuries, Exercise, Cultural equivalence, Cultural adaptation, Questionnaire, Physical activity

## Abstract

**BACKGROUND:**

The Hip Sports Activity Scale (HSAS) is a reliable and valid tool for determining the levels of sports activities among patients with femoroacetabular impingement (FAI).

**OBJECTIVE:**

To translate and cross-culturally adapt the HSAS to the Brazilian Portuguese language.

**DESIGN AND SETTING:**

This was a cross-sectional study conducted at the State University of Rio de Janeiro.

**METHODS:**

The Brazilian version of the HSAS was developed following a process that comprised six steps: translation, synthesis, back-translation, review by committee, pretesting and submission of documentation to the developers. The translation phase involved three independent bilingual translators whose mother language was Brazilian Portuguese. The back-translation phase involved three independent translators whose mother language was English. In order to verify comprehension of the questionnaire, 30 undergraduate students in physical education (65% men), with mean age 23.2 years (standard deviation = 6.8), participated in the pre-testing phase.

**RESULTS:**

During the translation step, some terms and expressions were changed to obtain cultural equivalence to the original HSAS. In the pre-testing phase, each item of the scale showed a comprehension level of 100%.

**CONCLUSION:**

The HSAS was translated from English to the Brazilian Portuguese language and adapted to Brazilian culture. The HSAS validation is ongoing.

## INTRODUCTION

There is growing evidence that femoroacetabular impingement (FAI) plays an important role in the mechanical etiology of the development of hip arthrosis. This abnormal contact between the acetabulum and the femoral neck during hip mobilization, especially during flexion and internal rotation, limits the range of motion.^1,4^ Impact can occur in patients who subject their hip to extreme ranges of motion, which can cause compression of the non-spherical extension of the supraphysiological head.^5^

Individuals with FAI complain mainly of chronic pain, with insidious onset, long duration and progressive worsening.^6^ Physical exercise generally causes exacerbations. In addition to sports activities, activities of daily living can also be associated with pain: for example, climbing stairs, sitting after a prolonged lying session, moving in bed and getting in and out of a car. The typical patient is a young adult, usually practicing sports that involve hip flexion. Pain can be constant, or intermittent at rest, and can interfere with sleep.^1,2,3,4,5,6,7^

Sports activities that require vigorous and repetitive flexion and internal hip rotation, such as ice hockey or football, are often associated with symptomatic FAI. In addition to these, martial arts such as kickboxing, taekwondo and kung-fu, and also speed athletics and hurdles, can be mentioned.

In this context, use of instruments to assess various aspects of health among individuals with different clinical conditions, in the form of questionnaires and scales, has proved to be very promising, based on patients’ perceptions of their health status.^8,9^

The Hip Sports Activity Scale (HSAS) is a reliable and valid tool for determining the levels of sports activities among patients who suffer femoroacetabular impingement. The original article was published by Naal et al. in 2013.^10^ Their research group has developed and validated a sports activity scale for patients with a diagnosis of femoroacetabular impingement, in English and German. The HSAS is composed of nine different levels of physical activity. It has nine items scored from 0 to 8, such that 0 represents sedentary individuals and 8 represents high-performance athletes, with no subscales. It has been widely used in English-speaking countries.

Many instruments have been developed in the English language.^11,12^ For them to be used in populations with different languages and cultures, it is necessary to follow a set of steps for their translation, cultural adaptation and validation, in order to ensure that the new instrument maintains the characteristics of the original version.^13,14^

## OBJECTIVE

The aim of this study was to translate the HSAS from English to the Brazilian Portuguese language and to cross-culturally adapt it to Brazilian culture. Our hypothesis was that the translation to Brazilian Portuguese and the cultural adaptation for use in Brazil would be feasible and acceptable.

## METHODS

### Type of study

This was a cross-sectional study of quantitative and qualitative nature on the translation and cross-cultural adaptation of a questionnaire, using data obtained between December 2014 and June 2015.

This study was approved by the ethics committee of our institution (number 998.832; date: March 15, 2015) and all subjects signed an informed consent statement. Dr. Florian D. Naal (the first author of the HSAS) gave permission for us to translate and cross-culturally adapt the HSAS to the Brazilian Portuguese language.

### Translation and cross-cultural adaptation

**Figure 1.**
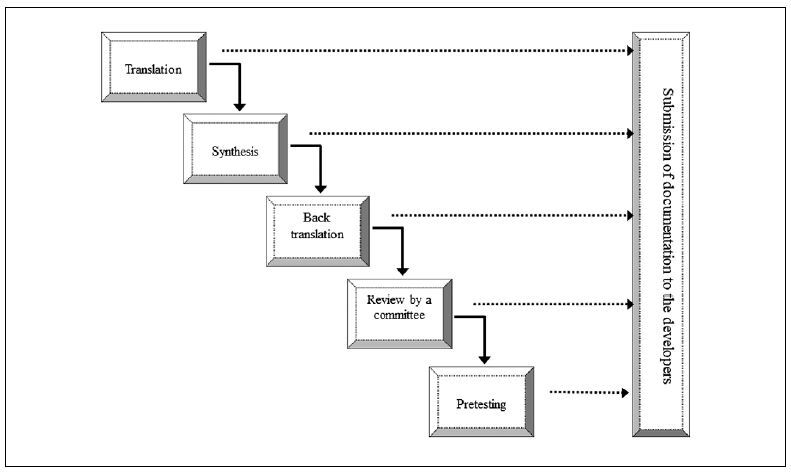
Steps of translation and cross-cultural adaptation according to the guidelines of Guillemin et al. and Beaton et al.: translation, synthesis, back-translation, review by a committee, pretesting and submission of documentation to the developers.^13,14^

To translate and adapt the HSAS, the guidelines suggested by Guillemin et al.^13^ and reviewed by Beaton et al.^14^ were followed. The process comprised six steps: translation, synthesis, back-translation, review by committee, pretesting and submission of documentation to the developers (Figure 1).

#### Translation (into the Brazilian Portuguese language)

The original English version of the HSAS was translated from English to Brazilian Portuguese by three independent translators (two physiotherapists and one orthopedist) with experience of hip treatment. They were informed about the purpose of the study. Three different Brazilian Portuguese translations were produced: T1, T2 and T3.

#### Synthesis (of translations produced into the Brazilian Portuguese language)

A multidisciplinary committee composed of two physiotherapists, three orthopedists and two physical educators was formed to evaluate these three initial Brazilian Portuguese translations (T1, T2 and T3) that were produced in the translation step. Possible distortions and their applicability were analyzed. A synthesis (S1) of these three Brazilian Portuguese translations was produced.

#### Back-translation (into the English language)

This Brazilian Portuguese synthesis (S1) was then back-translated into English by three independent translators who were unaware of the purpose of the translation. Three different back-translations into English were produced: BT1, BT2 and BT3.

#### Review by a committee (revised translation into the Brazilian Portuguese language)

The multidisciplinary committee that had been formed for back-translation reviewed these three back-translations into English (BT1, BT2 and BT3), in comparison with the original HSAS in English, and a revised translation into the Brazilian Portuguese language (RT) was developed. During this step, this committee verified the semantic, idiomatic, cultural and conceptual equivalences, in order to carry out the process of cultural adaptation for Brazil.

#### Pretesting (cultural equivalence)

The objective of this step was to assess situations, issues or terms that were not well understood. Thirty undergraduate students of physical education gave responses to the revised translation (RT) that had been produced in the review by the committee. No instructions to indicate whether there was an unknown term or sport, or to suggest sports that were not present on the scale, were given to the participants. We found that there were no questions with an incomprehension rate of more than 15%, and the students did not consider any of the questions to be not applicable. Based on these results, this revised translation (RT) was considered to be the final translation of HSAS.

#### Submission of documentation to the developers

The last step of the adaptation process was to send all the reports and forms to the developers of the translated version of the instrument.

## RESULTS

The sociodemographic characteristics of the 30 participants are shown in Table 1. Sixty-five percent of the volunteers were male, aged between 18 and 45 years, and 100% had completed high school education.

**Table 1.** Sociodemographic data on the thirty volunteers for pretesting

Volunteers for pretesting		
Gender	Female	20
	Male	10
Age (years)	Mean (standard deviation)	23.2 (6.8)
Marital status	Married	2
	Single	28
Education	High school not completed	0
	High school completed	30

Table 2 shows the changes made through verifying the semantic, idiomatic, cultural and conceptual equivalences, in order to carry out the cultural adaptation of the HSAS for use in Brazil.

**Table 2.** Modifications made to the Hip Sports Activity Scale (HSAS) within the cross-cultural adaptation for Brazil

Original HSAS	Modified to
Ice hockey and field hockey	Hockey
Snowboarding	Wakeboarding
Skiing	Surfing
Lacrosse	Removed
Cross-country skiing/Biathlon	
Cricket	
Racketball	
Badminton	

After these changes had been made by the multidisciplinary committee (at the step of review by a committee), the revised translation into Brazilian Portuguese language (RT) was produced.

In the pretesting, thirty volunteers, undergraduate students of physical education, gave responses to the RT. The results showed that there were no questions with an incomprehension rate of more than 15%, and the students did not consider any of the questions to be not applicable. From this result, our assessment was that the RT of the HSAS was well understood. Thus, the RT was considered to be the final translation of the HSAS, i.e. the HSAS-Brazil.

The original HSAS and the HSAS-Brazil adaptations are shown in **Box 1**. The HSAS-Brazil scale is available for download in **Annex 1**.

**Box 1.** The original Hip Sports Activity Scale (HSAS) and the Brazilian version (HSAS-Brazil)

Hip Sports Activity Scale (HSAS – English version)	Escala de Atividade Esportiva do Quadril (HSAS-Brasil)
**Please mark in the following list your current highest level of sports or recreational activity.**	**Por favor, marque na lista a seguir o mais alto nível de atividade esportiva ou recreacional atual que você consegue realizar.**
**8. Competitive Sports** ***(elite level)*** Soccer, Ice hockey, Field hockey, American football/Rugby, Martial arts, Tennis, Track-and-field, Indoor sports*, Beach-Volleyball, Lacrosse, Baseball/Softball	**8. Esportes de Competição** ***(nível elite)*** Futebol, Hóquei, Futebol americano/Rugby, Artes marciais, Tênis, Atletismo, Esportes de quadra*, Vôlei de praia, Beisebol/Softbol
**7. Competitive Sports** ***(elite level)*** Downhill skiing, Snowboarding **Competitive Sports** ***(minor leagues/collegiate)*** Soccer, Ice hockey, Field hockey, American football /Rugby, Martial arts, Tennis, Track-and-field, Indoor sports*, Beach-Volleyball, Lacrosse, Baseball/Softball	**7. Esportes de Competição** ***(nível elite)*** Surfe, Wakeboard **Esportes de Competição** ***(ligas menores/estudantil)*** Futebol, Hóquei, Futebol americano/Rugby, Artes marciais, Tênis, Atletismo, Esportes de quadra*, Vôlei de praia, Beisebol/Softbol
**6. Competitive Sports** ***(elite level)*** Golf, Bicycle racing, Mountain biking, Swimming, Rowing, Cross-country skiing/Biathlon, Horseback riding, Cricket **Competitive Sports** ***(minor leagues/collegiate)*** Downhill skiing, Snowboarding	**6. Esportes de Competição** ***(nível elite)*** Golfe, Ciclismo, Mountain bike, Natação, Remo, Hipismo **Esportes de Competição** ***(ligas menores/estudantil)*** Surfe, Wakeboard
**5. Competitive Sports** ***(minor leagues/collegiate)*** Golf, Bicycle racing, Mountain biking, Swimming, Rowing, Cross-country skiing/Biathlon, Horseback riding, Cricket **Recreational Sports** Soccer, Ice hockey, Field hockey, American football/Rugby, Martial arts, Track-and-field, Beach-Volleyball, Lacrosse	**5. Esportes de Competição** ***(ligas menores/estudantil)*** Golfe, Ciclismo, Mountain bike, Natação, Remo, Hipismo **Esportes Recreativos** Futebol, Hóquei, Futebol americano/Rugby, Artes marciais, Tênis, Atletismo, Vôlei de praia
**4. Recreational Sports** Tennis, Downhill skiing, Snowboarding, Indoor sports*, Baseball/Softball	**4. Esportes Recreativos** Tênis, Surfe, Wakeboard, Esportes de quadra*, Beisebol/Softbol
**3. Recreational Sports** Aerobics, Jogging, Lower extremity weight-training, Horseback riding, Cricket	**3. Esportes Recreativos** Ginástica aeróbica, Corrida, Musculação para membros inferiores, Hipismo
*2. Recreational Sports* *Golf, Bicycle racing, Mountain biking, Swimming, Rowing, Cross-country skiing/Biathlon, Dancing, Inline skating*	*2. Esportes Recreativos* *Golfe, Ciclismo, Mountain bike, Natação, Remo,* *Dança, Patinação*
**1. Recreational Sports** Swimming, Cycling, Hiking, Nordic walking (quick walking with ski-poles)	**1. Esportes Recreativos** Natação, Andar de bicicleta, Caminhada em trilhas, Caminhada em alta velocidade.
**0. No Recreational or Competitive Sports**	**0. Nenhum Esporte Recreativo ou de Competição**
*Indoor Sports: Basketball, Squash, Racketball, Handball, Badminton, Volleyball **Please indicate your preferred sport:** _____________________________.	*Esportes de Quadra: Basquete, Squash, Handebol, Vôlei **Por favor, indique seu esporte preferido:** _________________________.

## DISCUSSION

The HSAS was translated into the Brazilian Portuguese language and was cross-culturally adapted into Brazilian culture, thus confirming our hypothesis that adaptation of this scale for use in Brazil was indeed feasible and acceptable.

Guillemin et al.^13^ and Beaton et al.^14^ suggested in their guidelines that at least two translations of the original questionnaire or scale into the target language should be produced. In our study, we chose to perform three translations into Brazilian Portuguese language (T1, T2 and T3) and, consequently, three back-translations into English (BT1, BT2 and BT3) were produced. In the cross-cultural adaptations of the Harris Hip Score (HHS),^15^ the International Knee Documentation Committee (IKDC),^16^ the Nonarthritic Hip Score (NAHS)^17^ and the Hip Outcome Score (HOS)^18^ questionnaires, only two translations and two back-translations were performed for each of them. We believe that these three translations and three back-translations carried out by our group helped to produce a more careful and refined version of the HSAS-Brazil.

Questionnaires and scales developed in a foreign language need a careful cross-cultural adaptation process in order to allow them to be used in another sociocultural reality.^13^ The aim of the cross-cultural adaptation is to ensure consistency in content validity between the versions of the questionnaire (original language and target language). Subtle differences in life habits between different cultures can make an item on a questionnaire or scale more or less difficult to understand, thus changing the psychometric and statistical properties of the instrument.^14^ In our study, we decided to change the structure of the original instrument as little as possible: the changes made were extremely necessary for the process of adapting the HSAS to Brazilian culture.

“Ice hockey” and “field hockey” were considered to be “hockey” without discrimination between ice hockey and field hockey, since Brazilians do not usually practice ice hockey. As Brazilians are not used to “Nordic walking”, it was switched to “walking at high speed”. “Downhill skiing” and “snowboarding” were replaced by “surfing” and “wakeboarding”, water sports that are relatively popular in Brazil and because the skiing body movement is similar to that performed in surfing. In the cultural adaptation of the IKDC questionnaire, Metsavaht et al.^16^ also changed “skiing” to “surfing” because of the popularity of this sport in Brazil and the similarity of the stress applied to the knees while practicing these two sports activities.

“Cross-country skiing” and “cricket” were suppressed as they are not practiced in Brazil and because it was not possible to find an equivalent sport for them. “Biathlon” was also suppressed because it is included in cycling and swimming. “Badminton” was suppressed because it is not popular in Brazil; and, lastly, “racketball” was also suppressed because Brazilians seem to consider that this is the same as “squash”.

These changes were approved by the main author of the HSAS and these adaptations for the Brazilian version (HSAS-Brazil) are shown in **Box 1**.

In the pretesting, thirty volunteers who were undergraduate students of physical education gave responses to the revised HSAS translation into Portuguese (RT), so that we could assess comprehension of the scale and the semantic, idiomatic, cultural and conceptual equivalences. It was observed that other cultural adaptation studies also applied pretesting to a similar number of patients. Oliveira et al.^18^ applied pretesting of the HOS questionnaire to 30 patients with hip pain without arthrosis. Guimarães et al.^15^ administered pretesting of the HHS questionnaire to 30 patients with hip disorders. Del Castillo et al.^17^ performed pretesting of the NAHS questionnaire among 10 patients with hip pain and 20 healthy adults without hip pain.

One limitation of this study may have been the fact that the group of individuals who underwent the pretesting did not correspond to the target population of the scale, i.e. patients suffering from femoroacetabular impingement. However, for most questionnaires, the translations were not culturally adapted among individuals in their target population.^13^ Another limitation may have been the fact that the pretesting group was formed by undergraduate students, which does not reflect the general level of education of the Brazilian population. According to the Brazilian Institute for Geography and Statistics (Instituto Brasileiro de Geografia e Estatística, IBGE), half of all Brazilians have only attended school up to completion of elementary school.^19^

The strength of this study was its use of this tool to determine the levels of physical and sports activity, which is essential for evaluating younger patients, who tend to be physically very active. Their level of physical activity and participation in sports activities is an important prognostic factor. In addition, the pre-existing level may be directly related to the expectations desired by the patient. In this way, the HSAS contributes to filling the gap that existed among the questionnaires and scales for assessing the physically active population that suffers from femoroacetabular impingement.

## CONCLUSION

The Hip Sports Activity Scale was translated into the Brazilian Portuguese language and adapted to Brazilian culture. Our hypothesis that use of this scale in the Brazilian Portuguese language and Brazilian culture would be feasible and acceptable was found to be true. The validation process on the Hip Sports Activity Scale in Brazil is ongoing.

## Annex 1.

**Table:** The Brazilian version of the Hip Sports Activity Scale, HSAS-Brasil (Escala de Atividade Esportiva do Quadril).

ESCALA DE ATIVIDADE ESPORTIVA DO QUADRIL (HSAS-BRASIL) Por favor, marque na lista a seguir o mais alto nível de atividade esportiva ou recreacional atual que você consegue realizar.
**8. Esportes de Competição** ***(nível elite)*** Futebol, Hóquei, Futebol americano/Rugby, Artes marciais, Tênis, Atletismo, Esportes de quadra*, Vôlei de praia, Beisebol/Softbol.
**7. Esportes de Competição** ***(nível elite)*** Surfe, Wakeboard. **Esportes de Competição** ***(ligas menores/estudantil)*** Futebol, Hóquei, Futebol americano/Rugby, Artes marciais, Tênis, Atletismo, Esportes de quadra*, Vôlei de praia, Beisebol/Softbol.
**6. Esportes de Competição** ***(nível elite)*** Golfe, Ciclismo, Mountain bike, Natação, Remo, Hipismo. **Esportes de Competição** ***(ligas menores/estudantil)*** Surfe, Wakeboard.
**5. Esportes de Competição** ***(ligas menores/estudantil)*** Golfe, Ciclismo, Mountain bike, Natação, Remo, Hipismo. **Esportes Recreativos** Futebol, Hóquei, Futebol americano/Rugby, Artes marciais, Tênis, Atletismo, Vôlei de praia.
**4. Esportes Recreativos** Tênis, Surfe, Wakeboard, Esportes de quadra*, Beisebol/Softbol.
**3. Esportes Recreativos** Ginástica aeróbica, Corrida, Musculação para membros inferiores, Hipismo.
**2. Esportes Recreativos** Golfe, Ciclismo, Mountain bike, Natação, Remo, Dança, Patinação.
**1. Esportes Recreativos** Natação, Andar de bicicleta, Caminhada em trilhas, Caminhada em alta velocidade.
**0. Nenhum Esporte Recreativo ou de Competição**
***Esportes de Quadra:** Basquete, Squash, Handebol, Vôlei. **Por favor, indique seu esporte preferido:_________________________________.**
